# Sulfuretin promotes osteoblastic differentiation in primary cultured osteoblasts and *in vivo* bone healing

**DOI:** 10.18632/oncotarget.12460

**Published:** 2016-10-04

**Authors:** Q-Schick Auh, Kyung-Ran Park, Hyung-Mun Yun, Hyun-Chang Lim, Ga-Hyun Kim, Dong-Sung Lee, Youn-Chul Kim, Hyuncheol Oh, Eun-Cheol Kim

**Affiliations:** ^1^ Department of Oral Medicine, School of Dentistry, Kyung Hee University, Seoul, Republic of Korea; ^2^ Department of Oral & Maxillofacial Regeneration, Graduate School, Kyung Hee University, Seoul, Republic of Korea; ^3^ Department of Oral and Maxillofacial Pathology, School of Dentistry and Research Center for Tooth & Periodontal Regeneration (MRC), Kyung Hee University, Seoul, Republic of Korea; ^4^ Department of Periodontology, School of Dentistry, Kyung Hee University, Seoul, Republic of Korea; ^5^ Department of Biomedical Chemistry, College of Health and Biomedical Science, Konkuk University, Chung-Ju, Republic of Korea; ^6^ Institute of Pharmaceutical Research and Development, College of Pharmacy, Wonkwang University, Iksan, Republic of Korea

**Keywords:** Osteoblasts, Differentiation, In vitro, In vivo, Osteogenesis, Signal pathways, Pathology Section

## Abstract

Although sulfuretin, the major flavonoid of *Rhus verniciflua* Stokes, has a variety of biological actions, its *in vitro* and *in vivo* effects on osteogenic potential remain poorly understood. The objective of the present study was to investigate the effects of sulfuretin on *in vitro* osteoblastic differentiation and the underlying signal pathway mechanisms in primary cultured osteoblasts and on *in vivo* bone formation using critical-sized calvarial defects in mice. Sulfuretin promoted osteogenic differentiation of primary osteoblasts, with increased ALP activity and mineralization, and upregulated differentiation markers, including ALP, osteocalcin, and osteopontin, in a concentration-dependent manner. The expression levels of Runx2, BMP-2, and phospho-Smad1/5/8 were upregulated by sulfuretin. Moreover, sulfuretin increased phosphorylation of Akt, mTOR, ERK, and JNK. Furthermore, sulfuretin treatment increased mRNA expression of Wnt ligands, phosphorylation of GSK3, and nuclear β-catenin protein expression. *In vivo* studies with calvarial bone defects revealed that sulfuretin significantly enhanced new bone formation by micro-computed tomography and histologic analysis. Collectively, these data suggest that sulfuretin acts through the activation of BMP, mTOR, Wnt/β-catenin, and Runx2 signaling to promote *in vitro* osteoblast differentiation and facilitate *in vivo* bone regeneration, and might be have therapeutic benefits in bone disease and regeneration.

## INTRODUCTION

Osteoporosis, the most common cause of age-related bone loss, is characterized by low bone mass, microarchitectural deterioration of bone tissue, and increased risk of fractures [1]. Osteoporosis is known to occur as a result of a decrease in bone formation by osteoblasts and an increase in bone resorption by osteoclasts [1]. Therefore, stimulation of bone formation and inhibition of bone resorption have been suggested as an important therapeutic approach for the prevention and/or treatment of osteoporosis or bone disease [2].

Osteoblasts play a crucial role in bone formation through their proliferation and differentiation, which confers marked rigidity and strength to the bone while still maintaining some degree of elasticity [3]. Recently, three-dimensional cellular scaffolds are receiving significant attention in bone tissue engineering to treat bone defects. The Three-dimensional mesh structures with growth factors such as BMPs may be a feasible way to increase osteoblast differentiation and bone tissue repair [4–6]. In addition, hormone replacement therapy (HRT) effectively prevents bone diseases and reduces the incidence of fractures ith undesirable side effects [7,8]. Thus, it would be most helpful to develop new, effective, natural products that promote bone formation as alternative treatments for metabolic bone disorders and for the maintenance of bone health.

The stem bark of *Rhus verniciflua* Stokes (RVS) is used as a traditional medicine in East Asia for the treatment of gastritis, stomach cancer, and arteriosclerosis [6]. Previous studies have reported that sulfuretin, a major flavonoid component isolated from the heartwood of RVS, exerts a variety of beneficial properties [9–12]. We recently demonstrated that sulfuretin possesses cyto-protective effects in tert-butyl hydroperoxide-induced hepatotoxicity and anti-inflammatory effects in macrophages [13,14]. Moreover, Protective effects of sulfuretin were reported in a rheumatoid arthritis model [11,12]. It was recently reported that sulfuretin induces osteoblast differentiation through activation of TGF-β signaling in mesenchymal C3H10T1/2 cells, preosteoblast MC3T3-E1 cells, and primary bone marrow cells [15].

However, the osteogenic potential of sulfuretin in *in vivo* bone repair and the underlying mechanism of sulfuretin action in primary cultured osteoblasts have not been elucidated. The aims of this study were to evaluate the effects of sulfuretin on *in vitro* osteoblastic differentiation of primary osteoblasts and the underlying signal mechanism and on bone regeneration in an *in vivo* calvarial bone defect model and compare these effects to treatment with recombinant human bone morphogenetic protein 2 (rhBMP-2).

## RESULTS

### Effects of sulfuretin on osteoblastic differentiation of primary osteoblasts

**Figure 1:**
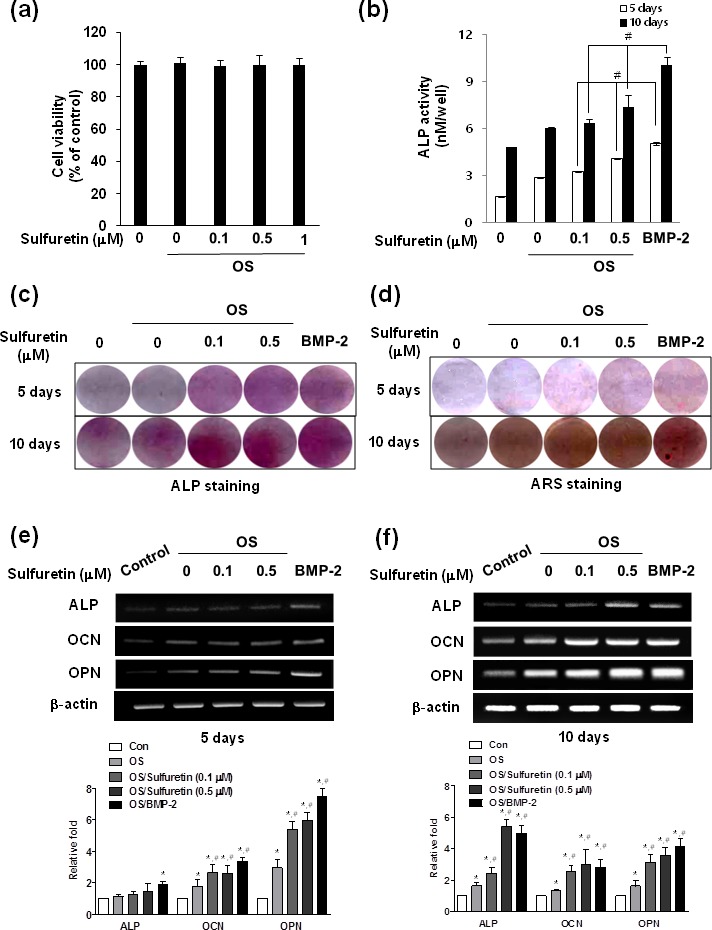
Effects of sulfuretin on cytotoxicity and osteoblastic differentiation in primary cultured osteoblasts (**A**) Cell viability was determined by the MTT assay. Differentiation was assessed by ALP activity (**B**), ALP or Alizarin red staining (**C**-**D**), and mRNA expression (**E**, **F**). Cells were cultured in osteogenic supplement medium (OS) containing 50 μg/ml L-ascorbic acid and 10 mM β-glycerophosphate with the indicated concentration of sulfuretin for 5 (A, B, E) or 10 days (B, C, F). Data (A, B) are presented as mean ± standard deviation (SD) (n = 3). *, *p* < 0.05 vs. control. #, *p* < 0.05 vs. each group. Graphs in E, F below are presented as mean ± standard deviation (SD) (n = 3). *, *p* < 0.05 vs. control. #, *p* < 0.05 vs. OS. The data shown are for n=3 in each category of sample in each experiment.

Before evaluating whether sulfuretin can stimulate osteoblast differentiation, its effect on cell growth was observed. As shown in Figure 1A, sulfuretin was not cytotoxic up to a concentration of 1 μM. To determine the anabolic activity of sulfuretin in bone metabolism, its effects on the activity of alkaline phosphatase ALP (an early marker of osteoblastic differentiation), mineralization (a late marker), and mRNA expression of various markers were evaluated in primary cultured osteoblasts. The results showed that sulfuretin increased ALP activity and matrix mineralization assessed by Alizarin red S staining in time-dependent manner (Figure 1B-1D). The mRNA expression of differentiation markers such as ALP, osteocalcin (OCN), and osteopontin (OPN) increased with sulfuretin treatment in a concentration- and time-dependent manner (Figure 1E, 1F). In addition, changes in ALP activity, mineralization, and mRNA expression with 0.5 μM sulfuretin were similar to those induced by the positive control (100 ng/mL rhBMP-2).

### Effects of sulfuretin on signal transduction pathways in primary osteoblasts

**Figure 2:**
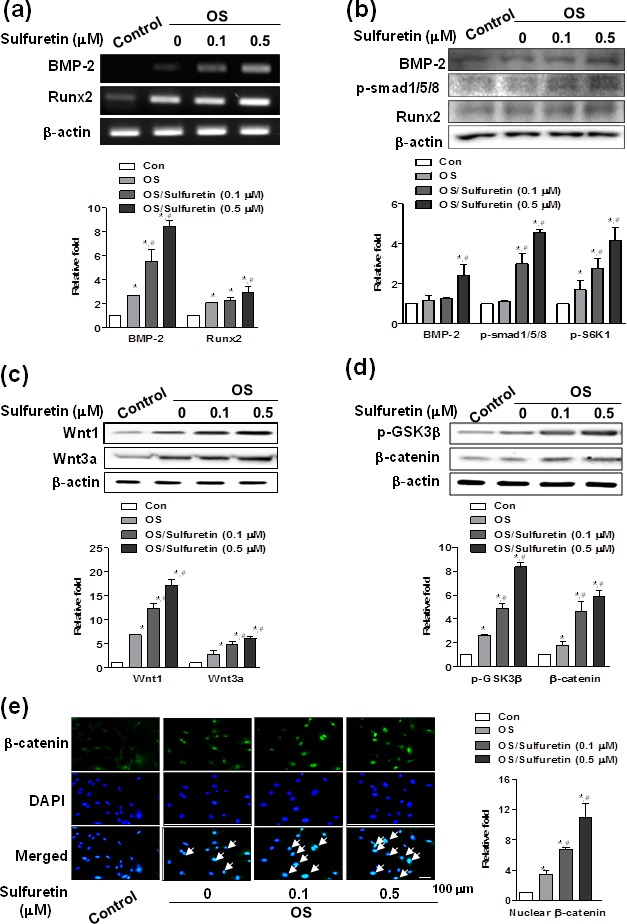
Effects of sulfuretin on the BMP and Wnt/β-catenin pathway in primary osteoblasts (**A**-**E**) Cells were cultured in osteogenic supplement medium with the indicated concentration of sulfuretin for 3 days (A, B) or 24 h (C-E). mRNA and protein expression were analyzed by RT-PCR (A), western blot (B-D), and immunocytochemistry (E) analysis, respectively. Similar results were obtained from three independent experiments. *, *p* < 0.05 vs. control. #, *p* < 0.05 vs. OS. The data shown are for n=3 in each category of sample in each experiment.

**Figure 3:**
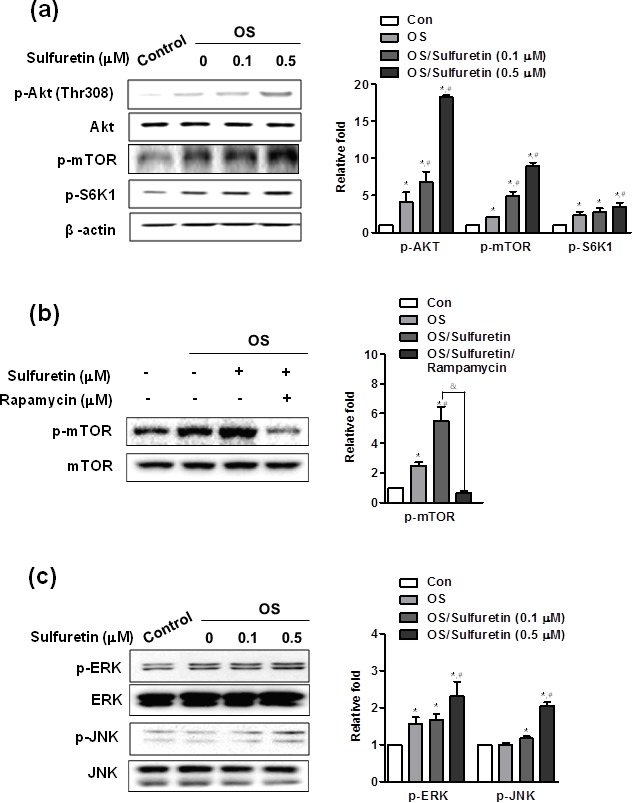
Effects of sulfuretin on the activation of Akt (A), mTOR (A, B), and MAP kinase (C) signaling in primary osteoblasts (**A**-**C**) Cells were cultured with osteogenic supplement and the indicated concentration of sulfuretin for 30 min (A, B) and 45 min (C). Where indicated, the cells were pretreated with rapamycin (20 nM) for 1 h and post-treated with 0.5 μM sulfuretin for 30 min (B). Protein expression was assessed by western blot analysis. *, *p* < 0.05 vs. control. #, *p* < 0.05 vs. OS. &, *p* < 0.05 vs. OS/sulfuretin. The data shown are for n=3 in each category of sample in each experiment.

To understand the signal regulatory mechanism of sulfuretin in the process of osteoblast differentiation, we evaluated the effects of sulfuretin on the activation of signaling molecules such as BMP, Wnt, Akt, mammalian target of rapamycin (mTOR), and mitogen-activated protein kinases (MAPK). Sulfuretin upregulated mRNA and protein expression of Runx2 and BMP-2, as well as phosphorylation of Smad 1/5/8, compared to the control (Figure 2A, 2B). Treatment with sulfuretin increased expression of the Wnt ligands Wnt1 and Wnt3a (Figure 2C). In addition, levels of phosphorylated glycogen synthase kinase-3β (GSK-3β) and β-catenin protein were increased by sulfuretin in a concentration-dependent manner, compared with the control (Figure 2D). This result was confirmed by the nuclear translocation of β-catenin detected by immunofluorescent staining (Figure 2E). In addition, phosphorylation levels of Akt and its downstream proteins mTOR and p70S6 kinase 1 (S6K1, a primary mTOR substrate) were increased in sulfuretin-treated osteoblasts (Figure 3A). Moreover, rapamycin, a selective mTOR inhibitor, attenuated the sulfuretin-induced phosphorylation of mTOR (Figure 3B). Since mTOR function is tightly regulated by the PI3K-Akt and MAPK signaling pathways, the phosphorylation levels of p38, ERK and JNK were examined. Phosphorylation of MAPK signaling proteins including ERK, and JNK was increased by sulfuretin in a concentration-dependent manner (Figure 3C), whereas that of p38 was not (data not shown).

### Effects of sulfuretin on bone regeneration in mouse critical-sized calvarial defect model

**Figure 4:**
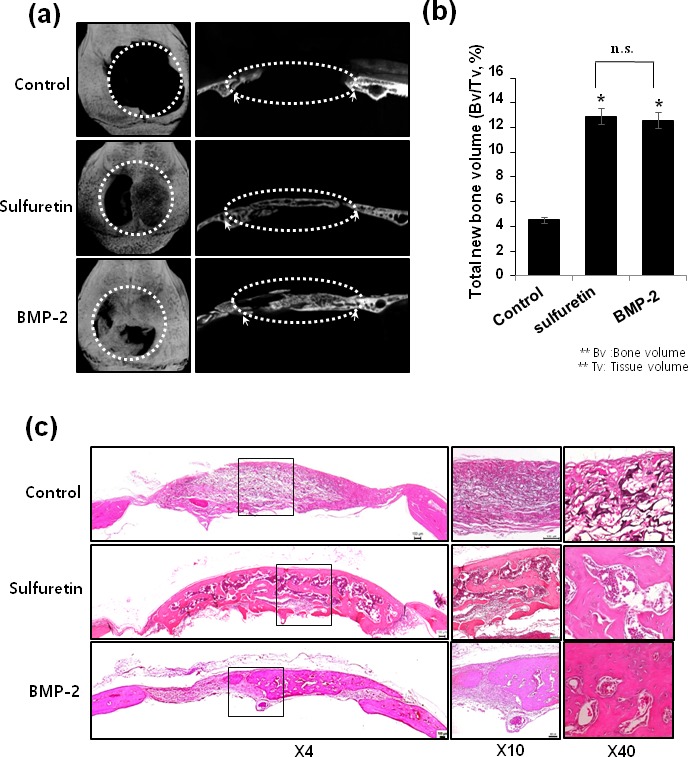
Effects of sulfuretin on *in vivo* bone regeneration (**A**) Representative micro-CT images of the calvarial defect at 6 weeks. (**B**) Quantitative analysis of new bone formation by 3D μCT. (**C**) Representative histologic images from H&E-stained calvarial bone defect (*left*) and higher magnification images (*right*). Datas (B) are presented as mean ± standard deviation (SD) (n = 6). The data shown are for n = 3 in each category of sample in each experiment. *, *p* < 0.05, vs. control, NS: non-significant.

To determine whether the sulfuretin-induced osteoblast differentiation and mineralization observed *in vitro* correlated with an increase in bone regeneration *in vivo*, we used the calvarial critical-sized defect animal model. 3D micro-computed tomography (μCT) showed that the negative control (Sham) had scant bone regeneration, whereas sulfuretin-treated groups had more bone regeneration than that of the control groups (Figure 4A). Densitometric μCT analysis revealed that the new bone volume fraction in the sulfuretin group was significantly increased compared with control groups (*P* < 0.05), and similar to the effects of treatment with rh-BMP2 (Figure 4B). For verification of bone healing, histologic examination was performed on decalcified calvarial defect samples. Control defects were filled with mostly fibrous tissue, but minimal bone healing was seen at the defect margins (Figure 4C). In the sulfuretin-treated defects, new bone formation and mature lamellar bone were observed at the margin and center of the defect. Similar bone regeneration with more mature bone at the defect was seen in the rhBMP-2-treated defects.

## DISCUSSION

In this study, we showed the stimulatory effects of sulfuretin on the differentiation of primary osteoblasts and related mechanisms *in vitro*, and then utilized a mouse calvarial defect model to explore the regulatory effects of sulfuretin on *in vivo* bone regeneration. We used primary osteoblasts isolated from calvaria of newborn animals for the *in vitro* studies because these cells are considered excellent sources of pure, active, and phenotypically mature osteoblasts as well as active progenitor cells without significant contamination with fibroblasts 16. Osteoblast cultures derived from fetal mice or rats are a commonly used model to analyze differentiation and mineralization *in vitro* [17, 18].

Our results demonstrated that sulfuretin induced high osteogenic and mineralization potential of primary osteoblasts in a dose- and time-dependent manner. The study showed a clear increase in ALP activity, mineralization, and expression of marker mRNAs with the use of sulfuretin. These measurements are indicative of induction of osteogenic differentiation parameters through the expression of early (ALP, ON), middle-stage (OPN), and late-stage (OCN and mineralization) markers [19]. Our investigation verified that the effects of 0.5 μM sulfuretin on ALP activity, mineralization, and mRNA expression were similar to the effects of 100 ng/mL rhBMP-2, which has been proven to stimulate osteoblast differentiation/mineralization and induce bone formation both *in vivo* and *in vitro* [20]. Our results showing that supplementation with sulfuretin (0.1-0.5 μM) stimulated osteoblastic differentiation in primary osteoblasts are consistent with previous reports for sulfuretin treatment (10-20 μM) in preosteoblastic MC3T3-E1 cells, C3H10T1/2 cells, and primary bone marrow cells [15].

The BMP pathway signaling is initiated by receptor binding, propagated by phosphorylation of the Smad1/5/8 complex, and finally translocated into the nucleus to regulate the transcription of target genes [21]. Among the downstream targets of BMPs is Runx2, which is the main transcription factor required for activation of osteoblast differentiation and is crucial for the regulation of genes responsible for the production of bone specific proteins such as OCN, OPN, and bone sialoprotein [22]. Our results showed that stimulation of primary osteoblasts with sulfuretin resulted in increased expression of Runx2 and BMP-2 mRNA and protein and Smad1/5/8 phosphorylation. These results suggested that one of the pathways activated by sulfuretin might be the BMP/Runx2 pathway.

Activated PI3K or Akt may positively regulate mTOR, leading to increased phosphorylation of ribosomal p70 S6 kinase (p70S6K) and eukaryotic initiation factor 4E binding protein 1 (4E-BP1), the two best-characterized downstream effector molecules of mTOR [23]. The PI3K/Akt/mTOR pathway has been shown to play important roles in the modulation of osteoblast functions in response to exogenous stimuli, including BMP [24, 25]. Our present study demonstrated for the first time that sulfuretin induces phosphorylation of Akt, mTOR, and p70S6K in primary osteoblasts, with Akt being upstream of mTOR/p70S6K. Pretreatment of osteoblasts with the mTOR inhibitor rapamycin inhibited the sulfuretin-induced increase in the phosphorylation of mTOR, indicating the importance of the mTOR pathway in modulating the differentiation of primary osteoblasts in response to sulfuretin.

The MAPK pathway has been shown to be important for mineralization and bone regeneration of osteogenic cells [26, 27, 28, 29]. Moreover, MAPK activation induces Runx2 phosphorylation and Runx2-dependent expression of the osteogenic genes [30]. In the present study, sulfuretin-treated osteoblasts showed activation of the ERK and JNK signaling pathways, which interplay with the BMP pathway in osteogenic differentiation [31]. It was recently demonstrated that MAPK regulates canonical Wnt/β-catenin signaling by inactivation of GSK-3β [32]. Canonical Wnt/β-catenin signaling is another pathway that regulates bone mass increase through a number of mechanisms such as osteoblastogenesis [33]. The canonical Wnt /β-catenin pathway is initiated by the binding of Wnt ligands, such as Wnt1 and Wnt3a, to the frizzled receptor and LRP5/6 coreceptor of the cell membrane, which inhibits GSK-3β activity and leads to accumulation of β-catenin in the cytosol and its subsequent translocation into the nucleus [34]. In the present study, expression of Wnt1, Wnt3a, p-GSK-3β, and nuclear β-catenin protein was increased in sulfuretin-stimulated osteoblasts in a concentration-dependent manner. Therefore, these data indicate that the Wnt/β-catenin pathway might play critical roles in the sulfuretin-promoted osteoblastic differentiation of osteoblasts.

In the present study, the mouse cranial defect was selected as the bone repair model because it has been widely used to assess the bone regeneration of bioactive molecules or biomaterials in bone sites [35, 36]. The μCT data demonstrated a significant increase in bone regeneration in defects treated with sulfuretin compared with the control group. Our μCT findings of increased new bone volume in the defects treated with sulfuretin correlated well with histologic analysis. This finding was further supported by the fact that rhBMP-2 treatment induced a similar increase in bone formation or regeneration by μCT and histologic analysis.

**Figure 5:**
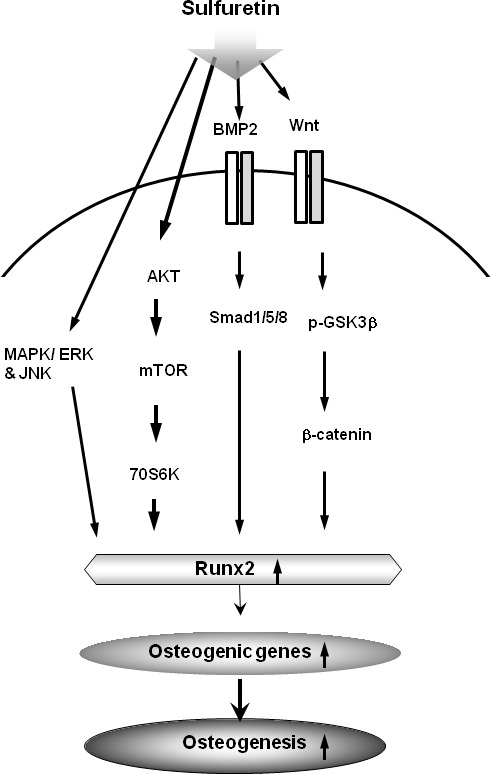
Schematic diagram illustrating that sulfuretin can induce osteoblastic differentiation and *in vivo* osteogenesis via activation of the BMP, mTOR, MAPK, and Wnt/β-catenin signaling pathways.

In summary, the present study demonstrated that sulfuretin promoted osteoblastic differentiation *in vitro* and significant bone regeneration *in vivo*. Schematic diagrams illustrating the potential mechanisms of sulfuretin signaling in primary osteoblasts and bone repair are collectively illustrated in Figure 5. Our findings suggest that sulfuretin may be a useful bone anabolic agent for the treatment of osteoporosis and bone tissue regeneration.

## MATERIALS AND METHODS

### Isolation and identification of sulfuretin

The heartwood of *Rhus verniciflua* Stokes (Anacardiaceae) was purchased from Dongbu Market, Iksan, Korea, in March 2009. The voucher specimen (WK-2009-42) was deposited at the Herbarium of College of Pharmacy, Wonkwang University (Iksan, Korea). Sulfuretin was isolated from the heartwood of *R. verniciflua* Stokes (Anacardiaceae) using a previously described method [14].

### Primary culture of mouse calvarial osteoblasts

Primary osteoblasts were isolated from calvariae of 1-day-old ICR mice after aseptic dissection and treated with 0.2% collagenase-dispase enzyme solution (Sigma-Aldrich, St. Louis, MO, USA). Cells from digestions 6–8 (10–25 × 10^6^ cells) were pooled and seeded at a density of 2 × 10^6^ cells/175 cm^2^ in culture flasks containing α-minimum essential medium (α-MEM) supplemented with 10% FBS and antibiotics (bensylpenicillin, gentamycin sulfate, and streptomycin). The cells were cultured for 4–6 days, with a change of medium every 2 or 3 days, at 37°C in a humidified atmosphere containing 5% CO2 in air. The experiments were approved by the Ethical Committee for Animal Experiments at Kyung Hee University (Seoul, Korea). To induce differentiation, cells were cultured with sulfuretin or rh-BMP2 (Calbiochem Co., La Jolla, CA, USA) and osteogenic supplement (OS; 50 μg/mL ascorbic acid and 10 mM β-glycerophosphate) as described previously [17].

### Alkaline phosphatase (ALP) activity

Incubated primary osteoblasts were washed with PBS and then sonicated with a cell disruptor. ALP activity was measured using p-nitrophenyl phosphate (3 mM final concentration) as the substrate in 0.7 M 2-aminomethyl-1-propanol (pH 10.3) and 6.7 mM MgCl_2_. The absorbance was measured at 405 nm using an enzyme-linked immunosorbent assay reader (Beckman Coulter, Fullerton, CA, USA).

### ALP and Alizarin red S staining

Primary osteoblasts were fixed in 70% ice-cold ethanol for 1 h and rinsed with distilled water. For Alizarin red staining, fixed cells were stained with 40 mM Alizarin Red S (pH 4.2) for 10 min with gentle agitation. The level of Alizarin Red S staining was observed under light microscopy. An ALP staining kit (86R-1KT, Sigma-Aldrich) was used according to the manufacturer's instructions.

### RT-PCR

Total RNA of cells was extracted with Trizol reagent (Life Technologies, Gaithersburg, MD, USA) according to the manufacturer's instructions. One microgram of total RNA was reversed transcribed to cDNA with a first strand cDNA synthesis kit using random primers (Bioneer, Daejeon, Korea) and the generated cDNAs were amplified with AccuPower PCR PreMix (Bioneer). The sequences of primers used were as follows: β-actin sense, AATGTGGCTGAGGACTTTG and antisense, GGGACTTCCTGTAACCACTTATT; Runx2 sense, ACTCTTCTGGAGCCGTTTATG and antisense, GTGAATCTGGCCATGTTTGTG; BMP2 sense, ACACAGCTGGTCACAGATAAG and antisense CTTCCGCTGTTTGTGTTTGG. The PCR products were electrophoretically size fractionated and analyzed on ethidium bromide-stained 2% agarose gels. PCR products were quantified using Histogram tool of Adobe Photoshop program and normalized by β-actin, a representative reference gene. The numeric values were calculated as relative fold of control.

### Western blot analysis

Cells were lysed and the protein concentration of lysates was determined using Bradford reagent (Bio-Rad, Hercules, CA, USA) as described previously [37]. Equal amounts of lysates were resolved by sodium dodecyl-polyacrylamide gel electrophoresis (SDS–PAGE) and transferred to a polyvinylidene fluoride (PVDF) membrane (Millipore). The membrane was blocked with 1× TBS containing 0.05% Tween 20 and 5% skim milk or 2% BSA for 1 h at room temperature and then incubated overnight at 4°C with the respective primary antibodies. The membranes were washed three times and incubated with diluted horseradish peroxidase (HRP)-conjugated secondary antibodies (1:10,000) for 1 h at room temperature. After three washes, the signals were detected using an enhanced chemiluminescence (ECL) kit (Amersham, Piscataway, NJ, USA)

### Immunocytochemistry

Cells were grown on glass coverslips and incubated with sulfuretin for 24 h. Cells were fixed in 10% formalin for 15 min at room temperature. After washing three times in 1× PBS, the cells were permeabilized with 0.2% Triton X-100 in 1× PBS for 20 min, washed three times in 1× PBS, and then blocked with 5% BSA in 1× PBS for 1 h at room temperature. The cells were incubated with anti-β-catenin (1:200, Abcam) antibodies overnight at room temperature, washed three times, and incubated with Alexa-488 conjugated secondary antibodies (1:500, Invitrogen, Carlsbad, CA, USA) for 2 h at room temperature. The cells were stained with DAPI (Sigma-Aldrich), washed three times, mounted on glass slides, and viewed by fluorescence microscopy (Nikon Instruments Inc., Melville, NY, USA).

### Surgical procedure for calvarial bone defects in mice

Female 8-week-old ICR mice (*Samtako*, Osan, Kyoung Gi-Do, Korea) used in this study were maintained in accordance with National Institute of Toxicological Research of the Korea Food and Drug Administration guidelines for the humane care and use of laboratory animals. All experimental procedures were approved by Kyung Hee University Animal Care Committee (approval number: KHMC-IACUC 2015-002). Surgical procedure for calvarial bone defects was performed as described previously38. The critical-sized defects in mice were randomly divided into three groups to receive the following surgery: (1) sham-surgery (empty) controls (*n* = 6); (2) sulfuretin (0.27 μg/g mouse weight, *n* = 6); (3) rh-BMP-2 (1 μg/g mouse weight, *n* = 6). Animals were sacrificed 6 weeks postsurgery and the calvarial bone was carefully excised, cleaned, and fixed immediately in 10% formalin. Tissues were decalcified in 10% EDTA for 14 days, embedded in paraffin, and sectioned at 5 μm. Sections were stained with hematoxylin and eosin (H&E).

### Micro-computed tomography (μCT)

Micro-CT was performed at the Advanced Institutes of Convergence Technology (Genoss Co., Ltd., Gyeonggi-do Korea) as described previously38. Micro-CT data of calvaria were acquired on a Skyscan 1173 scanner (Bruker-microCT, Kontich, Belgium). Realistic 3D-Visualization software (Bruker-microCT) was used to reconstruct the CT images three-dimensionally from data acquired on approximately 2,000 cross-sections.

### Statistical analysis

Data were analyzed using the GraphPad Prism version 4 program (GraphPad Software, Inc., San Diego, CA, USA) and are presented as mean ± standard deviation. Comparisons between groups were analyzed by *t*-tests (two-sided) or by ANOVA for experiments with more than two subgroups. Probability values of *P* < 0.05 were considered to be statistically significant.
